# Supplementary material for: Differentiation of Leishmania (L.) infantum, Leishmania (L.) amazonensis and Leishmania (L.) mexicana Using Sequential qPCR Assays and High-Resolution Melt Analysis
Source: Microorganisms. 2020 May 29;8(6):818. doi: 10.3390/microorganisms8060818 (PMC7355826; doi:10.3390/microorganisms8060818)
Supplement: Supplementary file 1 [file microorganisms-08-00818-s001.zip › supplementary files.docx]

**
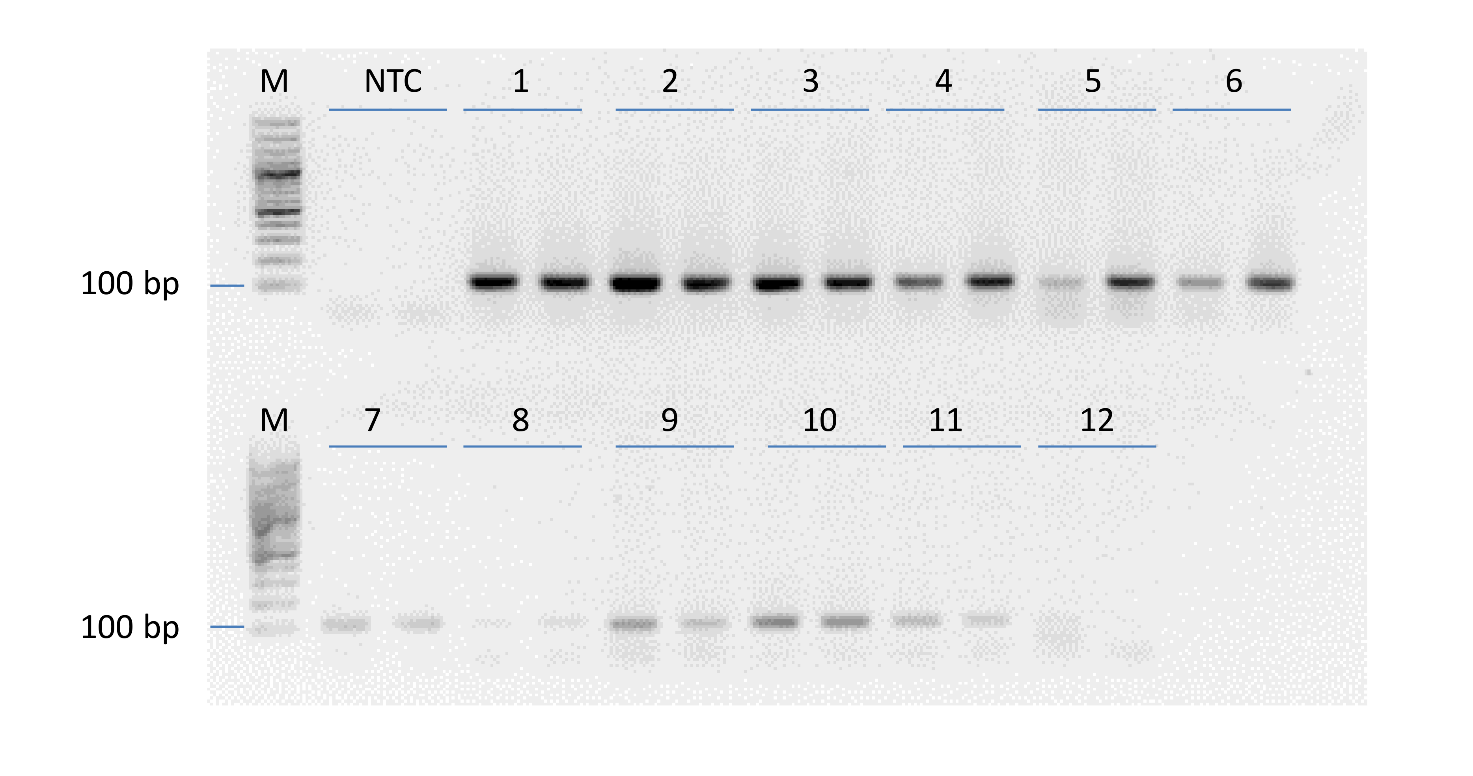
**

**Figure S1.** Electrophoretic analysis of qPCR-ML products. Lane 1: *L.* (*L.*) *infantum* MHOM/TN/80/IPT1; Lane 2: *L.* (*L.*) *infantum* MHOM/IT/86/ISS218; Lane 3: *L.* (*V.*) *braziliensis* MHOM/BR/75/M2904; Lane 4: *L.* (*L.*) *amazonensis* MHOM/BR/00/LTB0016; Lanes 5-8: *L*. (*L.*) *mexicana* MHOM/MX/2011/Lacandona serial dilution ranging from 10.0 to 0.01 ng DNA/reaction tube; Lanes 9-12: *L*. (*L.*) *mexicana* MHOM/MX/2011/Lacandona serial dilution ranging from 10.0 to 0.01 ng DNA/reaction tube spiked with 30 ng of human DNA. Abbreviations: M, 100 bp DNA ladder; NTC, no template control.

**
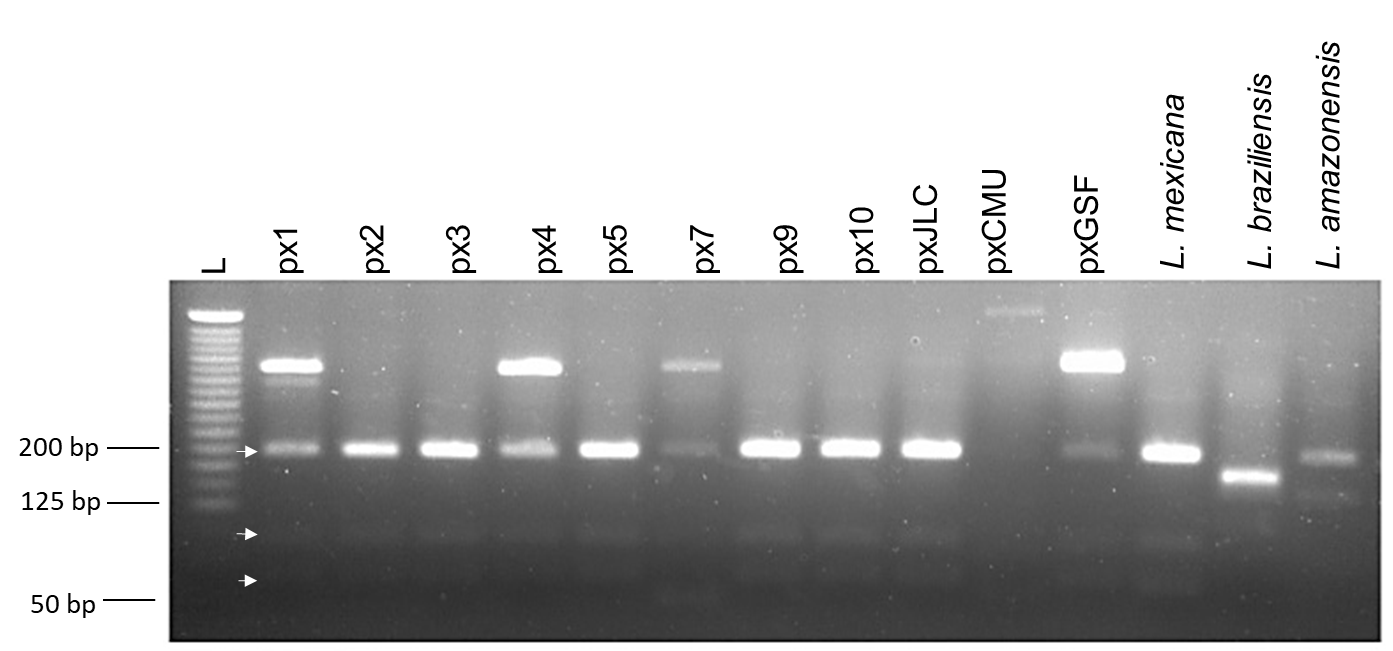
**

**Figure S2**. Digestion of ITS1 amplicons of clinical samples and reference *Leishmania* strains with the restriction endonuclease HaeIII. The following strains of *Leishmania* have been used as reference: *L. mexicana* MHOM/MX/2011/Lacandona, *L. braziliensis* MHOM/BR/75/M2904, *L. amazonensis* MHOM/BR/00/LTB0016. Fragments were separated on a 4% agarose gel. The fragments specific for *L. mexicana* are indicated by arrows. L: 25bp ladder.

**
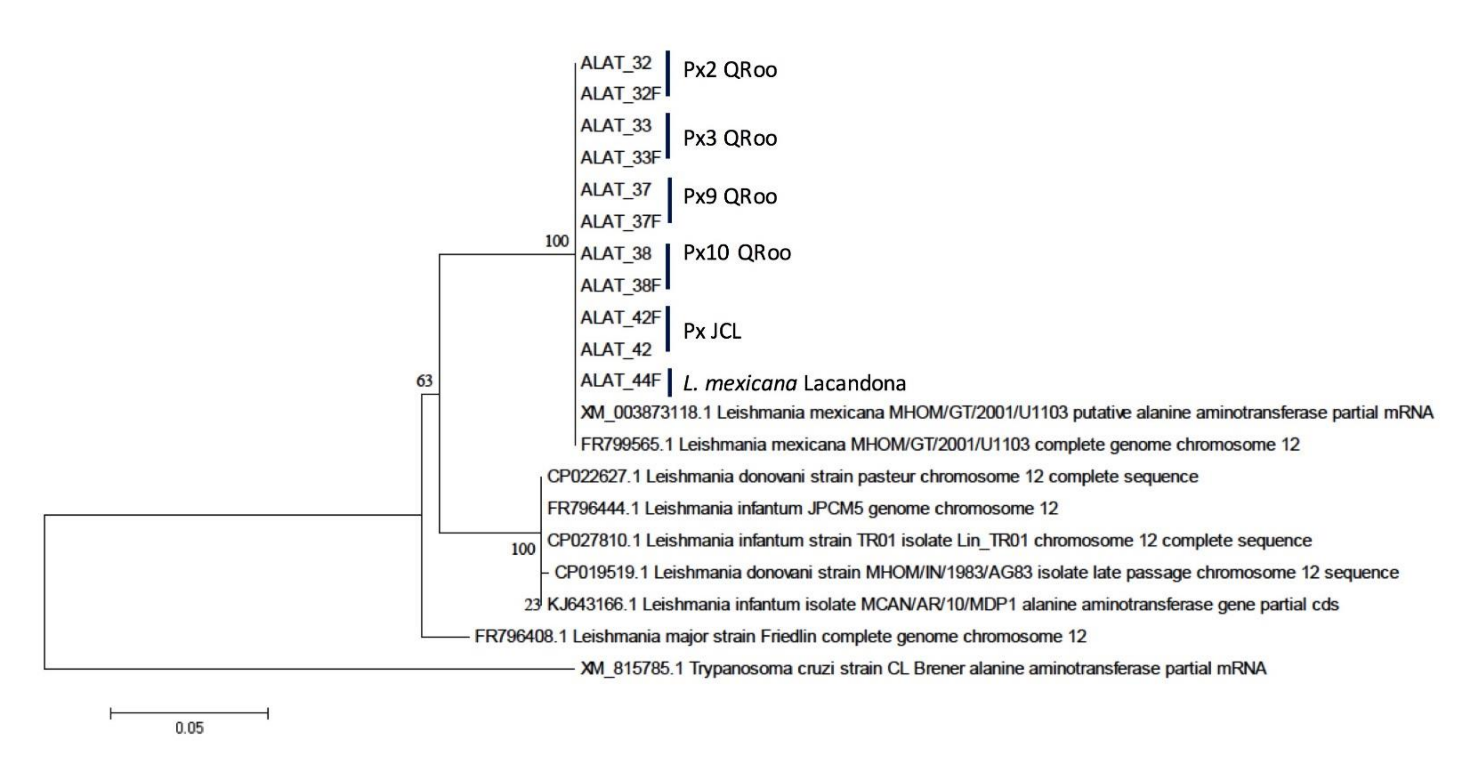
**

**Figure S3.** Maximum likelihood phylogenetic tree of ALAT amplicons. The tree was constructed with MEGA 6.0 software using the close neighbor interchange method.

**
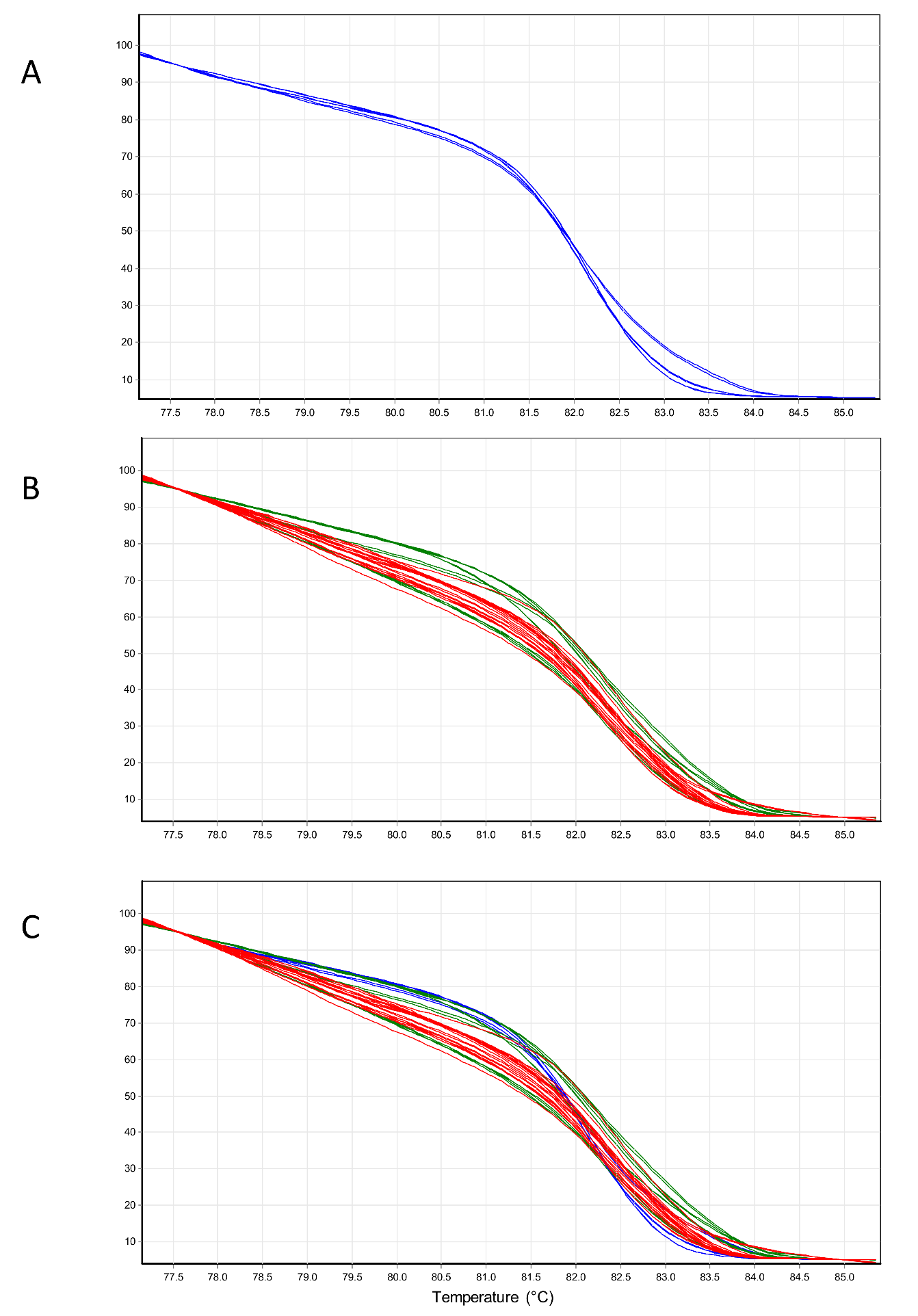
**

**Figure S4.** HRM analysis of qPCR-ama amplicons. A) Normalized HRM curves obtained from amplicons of *L*. (*L*.) *amazonensis* MHOM/BR/00/LTB0016, *L*. (*L*.) *amazonensis* clinical isolate and *L*. (*L*.) *amazonensis* IFLA/BR/67/PH8 (blue lines); B) Normalized HRM curves obtained from amplicons of *L*. (*L*.) *mexicana* isolates 2, 3, 5, 14, 17, MHOM/MX/2011/Lacandona (green lines) and *L*. (*L*.) *mexicana* clinical samples (n=11) (red lines); C) Merged curves from panels A and B. Each sample was tested in duplicate.

**Table S1** Results of qPCR-ITS1 HRM analysis from *L*. (*L*.) *amazonensis* and *L*. (*L*.) *mexicana* samples.

| Species | Strain/isolate/clinical sample | qPCR-ITS1  HRM (°C)±SD |
| --- | --- | --- |
| *L*. (*L*.) *mexicana* | MHOM/MX/2011/Lacandona | 79.90±0.028 |
| *L*. (*L*.) *mexicana* | Isolate 2 | 79.82±0.005 |
| *L*. (*L*.) *mexicana* | Isolate 3 | 79.82±0.006 |
| *L*. (*L*.) *mexicana* | Isolate 5 | 79.91±0.035 |
| *L*. (*L*.) *mexicana* | Isolate 14 | 79.89±0.014 |
| *L*. (*L*.) *mexicana* | Isolate 17 | 79.88±0.005 |
| *L*. (*L*.) *mexicana* | Px1 | n.a. |
| *L*. (*L*.) *mexicana* | Px2 | 79.88±0.007 |
| *L*. (*L*.) *mexicana* | Px3 | 79.85±0.005 |
| *L*. (*L*.) *mexicana* | Px4 | 79.93±0.078 |
| *L*. (*L*.) *mexicana* | Px5 | 79.85±0.005 |
| *L*. (*L*.) *mexicana* | Px7 | - |
| *L*. (*L*.) *mexicana* | Px9 | 79.78±0.007 |
| *L*. (*L*.) *mexicana* | Px10 | 79.80±0.005 |
| *L*. (*L*.) *mexicana* | PxGSF | - |
| *L*. (*L*.) *mexicana* | PxCMU | - |
| *L*. (*L*.) *mexicana* | PxJLC | 79.88±0.035 |
| *L. (L.) amazonensis* | MHOM/BR/00/LTB0016 | 79.48±0.035 |
| *L. (L.) amazonensis* | IFLA/BR/67/PH8 | 79.21±0.014 |
| *L. (L.) amazonensis* | Clinical isolate | 79.51±0.014 |

n.a.: not available

-: negative result
